# Overview of the therapeutic efficacy of marine fish oil in managing obesity and associated metabolic disorders

**DOI:** 10.14814/phy2.70019

**Published:** 2024-10-02

**Authors:** Riya Kar, Titli Panchali, Pipika Das, Ananya Dutta, Manisha Phoujdar, Shrabani Pradhan

**Affiliations:** ^1^ Biodiversity and Environmental Studies Research Center Midnapore City College, affiliated to Vidyasagar University Midnapore West Bengal India; ^2^ Central Research Laboratory, Department of Paramedical and Allied Health Sciences Midnapore City College Midnapore West Bengal India

**Keywords:** fat oxidation, marine FO, metabolic disorders, obesity, omega‐3 polyunsaturated fatty acids

## Abstract

In the present scenario, obesity is a challenging health problem and its prevalence along with comorbidities are on the rise around the world. Ingestion of fish becomes trendy in daily meals. Recent research has shown that marine fish oil (FO) (found in tuna, sardines, and mackerel) may offer an alternative method for reducing obesity and problems associated with it. Marine FO rich in long‐chain omega‐3 polyunsaturated fatty acids (LC n‐3 PUFA) and long‐chain omega‐6 polyunsaturated fatty acids (LC n‐6 PUFA) plays an important role in reducing abnormalities associated with the metabolic syndrome and has a variety of disease‐fighting properties, including cardioprotective activity, anti‐atherosclerotic, anti‐obesity, anti‐cancer, anti‐inflammatory activity. Studies in rodents and humans have indicated that LC n‐3 PUFA potentially elicit a number of effects which might be useful for reducing obesity, including suppression of appetite, improvements in circulation, enhanced fat oxidation, energy expenditure, and reduced fat deposition. This review discusses the interplay between inflammation and obesity, and their subsequent regulation via the beneficial role of marine FO, suggesting an alternative dietary strategy to ameliorate obesity and obesity‐associated chronic diseases.

## 1 INTRODUCTION

Recently obesity is becoming the pandemic of the 21st century and its prevalence rate growing very rapidly. Obesity is considered as a heavier problem of our society due to its associated complications (Ray et al., 2019). In spite of above statement as per 2021, Ministry of Statistic and Programme Implementation stated that total population of India is around 1.39 billion more than 135 million individuals are affected by obesity, however the prevalence rate of obesity is 40.3% (Venkatrao et al., 2020). Based on the 2023 World Obesity Atlas, obesity prevalence is expected to increase globally over the next 10 years (Peng et al., 2023). According to Ferreira et al. (2024), it should come as no surprise that obesity has gained prominence on the global public health agenda and has drawn funding from a variety of societal sectors in an effort to slow down this grave health issue. The metabolism of fat and glucose is greatly influenced by adipose tissue (AT), which is a primary location of energy storage. Adipokines, such as leptin, factor‐alpha, interleukin‐6, and monocyte chemoattractant protein‐1, are known to be expressed and secreted by AT (Antuna‐Puente et al., 2008). Due to the disruption of adipokine secretion from white adipose tissue (WAT), an excessive amount of WAT contributes to obesity and obesity‐related disorders like diabetes mellitus, hypertension, dyslipidemia, and cardiovascular disease in the current nutritionally abundant environment (Walker et al., 2007). Clinical practitioners occupy a crucial role in the front line of obesity and its related comorbidities management, however currently recommended therapies with evidence‐based support are lifestyle intervention, pharmacotherapy, and bariatric surgery (Lorente‐Cebrián et al., 2013). Only modest benefits have been seen from therapeutic approaches focused on dietary and lifestyle modifications. Pharmacotherapy typically boosts catabolism or improves satiety and suppresses hunger (Gjermeni et al., 2021). In modern times, several drugs, such as orlistat, lorcaserin, liraglutide, phentermine‐topiramate, are available in the market for the treatment of obesity and overweight patient (Cui et al., 2017) but these drugs are not free from side effects (Pradhan et al., 2020). Numerous studies stated that marine FO could be a fruitful substitute to reduce obesity and obesity‐related metabolic disorders (de Sá et al., 2016). Fish is regarded as a budget‐friendly source of a wide range of beneficial elements, mostly high in fat and protein, both of which are important components of a human diet. Fish protein is particularly vital for muscle tissue growth and repair, as well as for immune system enhancement (Pal et al., 2018). The oil obtained from fish contain triglycerides, phospholipids, fatty acids, wax esters, sterols, other minor compounds like glyceryl esters, glycolipids, hydrocarbons like squalene, sulfolipids. While phospholipids and sterols are structural elements of the cell membrane, other lipids function as energy reserves. Marine FO contains a more diverse range of fatty acids, with a particular emphasis on polyunsaturated and monounsaturated fatty acids (PUFA and MUFA) (Das et al., 2024). Dietary sources of long‐chain polyunsaturated fatty acids (LC n‐3 PUFAs), such as eicosa pentaenoic acid (EPA) and docosahexaenoic acid (DHA), from marine FO have been shown to have hypotriglyceridemic, anti‐inflammatory, and cardioprotective effects (Carpentier et al., 2006). Thus, it is particularly interesting to see how marine FO (LC n‐3 PUFAs) affects body weight and body composition. We provide an update on the impact of marine FO (LC n‐3 PUFAs) on obesity and its related disorders in this review, emphasizing the possible mechanisms for LC n‐3 PUFAs in improving body composition, lowering body weight, and mitigating the negative metabolic effects of obesity.

## 2 BASIC PATHOMECHANISM OF OBESITY

**FIGURE 1:**
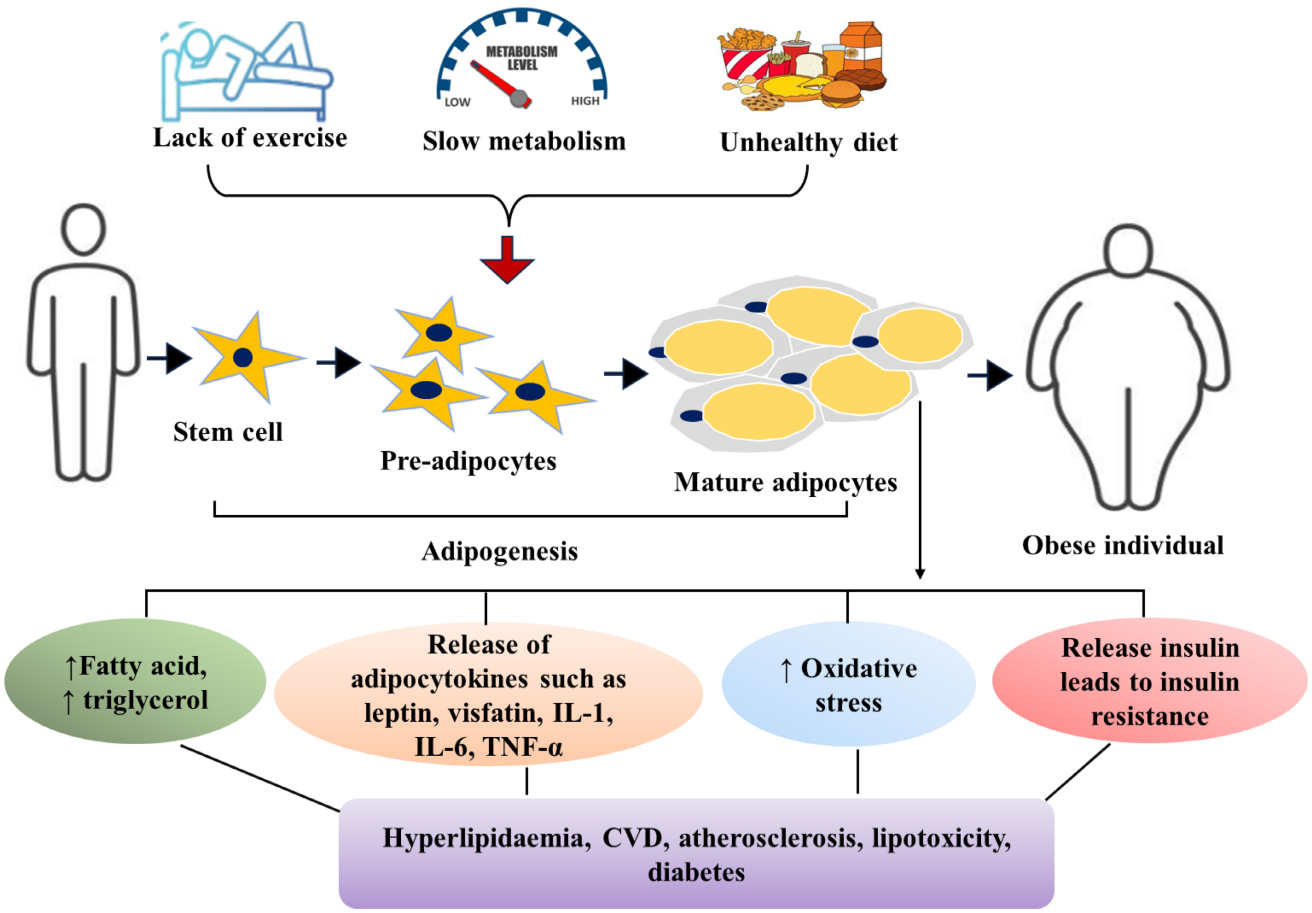
Overview of alterations in AT with obesity‐associated adipogenesis that responsible for release of some adipocytokines, free fatty acid, triglycerol, and raised the risk of several metabolic disorders in an obese individual.

The basic pathogenesis of obesity involves controlling cellular processes, physical activity, and other factors to either upregulate appetite or downregulate calorie utilization. This dysregulation causes an abundance of adipocytes to form, which boosts the release of cytokines and causes the development of vascular complications. The excess blood level of fatty acids and triglycerol causes accumulation of adipocytes which leads to an increase in oxidative stress, hypertriglyceridemia, lipotoxicity, diabetes, and various metabolic syndromes. Thus, lowering oxidative stress, which is a common etiological factor in many pathological conditions, may be advantageous in minimizing the risky effects of obesity and other complications (Karri et al., 2019). The adipocytes are responsible to stimulating the release of adipocytokines, such as leptin, adiponectin and vastatin, where adiponectin may responsible for cytotoxic autophagy in breast‐, colon‐, prostate‐, and female‐specific carcinogenesis (Muppala et al., 2017). These hormones regulate hunger, satiety, and body fat, and their dysregulation can lead to obesity (Karri et al., 2019) (Figure 1).

## 3 HORMONAL REGULATION IN OBESITY THROUGH ENERGY BALANCE

Compared to lean individuals, obese people exhibit a different hormonal profile, which may affect energy balance and contribute to maintaining a higher weight. Hormonal and metabolic alterations take place in response to diet‐induced weight loss that upsets this energy balance equilibrium (Sumithran et al., 2011).

### 3.1 Energy imbalance in obesity

**FIGURE 2:**
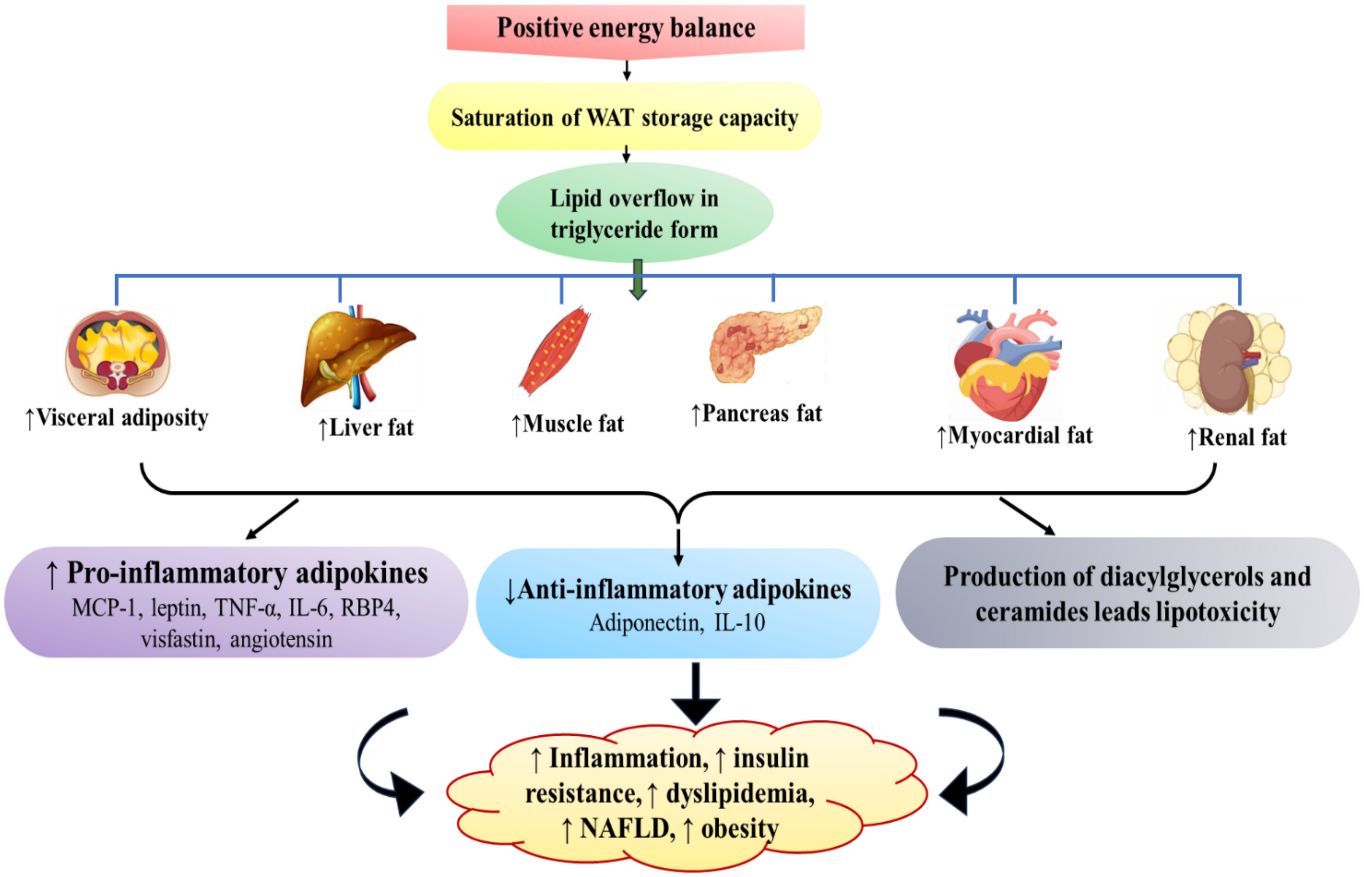
Contribution of WAT to obesity and its associated metabolic complications via lipotoxicity, upregulation, and downregulation of some adipokines. Due to alteration of positive energy balance, excessive lipid accumulated in the peripheral organs that leads tissue damage.

Energy intake, expenditure, and storage are the three main elements of an energy balance. The only time that a person's body weight can change is when their daily energy intake is greater than their daily energy expenditure (Bray & Bouchard, 2014). After increasing body weight when the storage capacity of white AT became saturated, lipid accumulation started in triglycerides from our peripheral organs (liver, muscle, pancreas etc.) and the excess fat enter into the non‐oxidative pathway. Throughout this process, toxic reactive lipid species, such as diacylglycerols, ceramides, are produced and ultimately causes tissue damage as well as lipotoxicity (Cao, 2014) and side by side upregulate the expression of pro‐inflammatory adipokines, downregulate the expression of anti‐inflammatory adipokines that ultimately leads to develop obesity and obesity‐associated disorders (Figure 2).

### 3.2 Long‐acting hormonal regulation in energy balance

It has been demonstrated that a number of hormones can influence satiety in both humans and animals by directly affecting the central nervous system (CNS) and the peripheral nervous system. According to Gjermeni et al. (2021), leptin and insulin are the primary players in controlling energy balance as a result of long‐term energy reserves. Leptin's physiological function is to convey nutritional status, especially low energy. Thus, a decrease in leptin levels is linked to fasting and losing weight (Busebee et al., 2023). Leptin binds to receptors in the brainstem, hypothalamus, and arcuate nucleus which express pro‐opiomelanocortin (POMC) to affect hunger and calorie consumption. POMC undergoes posttranslational processing to produce melanocortin peptides (alpha, beta, and gamma MSH), which act as agonists for melanocortin 3 and 4 receptors. By a variety of melanocortin‐dependent and ‐independent mechanisms, leptin signaling affects energy balance. Energy intake and expenditure are regulated by these hypothalamic pathways in conjunction with other brain regions. Severe obesity is caused by homozygous loss‐of‐function mutations in POMC that affect signaling through the leptin receptor or reduce leptin production, secretion, or biologic activity. According to studies, targeted genetic disruption of MC4R in mice results in increased food intake, increased lean mass, and linear development, characteristics that completely coincide with those reported in humans with loss‐of‐function mutations in MC4R (van der Klaauw & Farooqi, 2015). Hormones linked to feeding circulate during the process of food digestion and absorption, which can impact feelings of fullness and the need to stop eating. After eating, and especially after consuming glucose, insulin is secreted. There seems to be a hunger response associated with this postprandial raise in insulin. Throughout the brain, insulin receptors are present, and insulin can pass the blood–brain barrier (Honda et al., 2007).

### 3.3 Short‐acting hormonal regulation in positive energy balance: Orexigenic

Dietary consumption, utilization of energy, and metabolism of glucose all are regulated by POMC, agouti‐related peptide, and neuropeptide Y (AgRP/NPY) neurons located in the arcuate nucleus. Insulin activates AgRP/NPY and POMC neurons, which carry insulin receptors (Belfort‐DeAguiar & Seo, 2018). These are basically antagonistic pathways that converge at targets such as cells in the paraventricular nucleus of the hypothalamus that express melanocortin MC4R. The POMC neurons appear to trigger MC4R‐expressing cells in the paraventricular nucleus, which induce satiety. Conversely, the orexigenic hormone ghrelin is mostly released by the stomach (Date et al., 2000). According to Higgins et al. (2007), ghrelin levels rise before a meal and then fall to their baseline levels thereafter. Furthermore, ghrelin induces the release of growth hormone. It causes changes in many gastrointestinal functions and increases food intake by stimulating the vagus nerve.

### 3.4 Short‐acting hormonal regulation in negative energy balance: Anorexigenic

**FIGURE 3:**
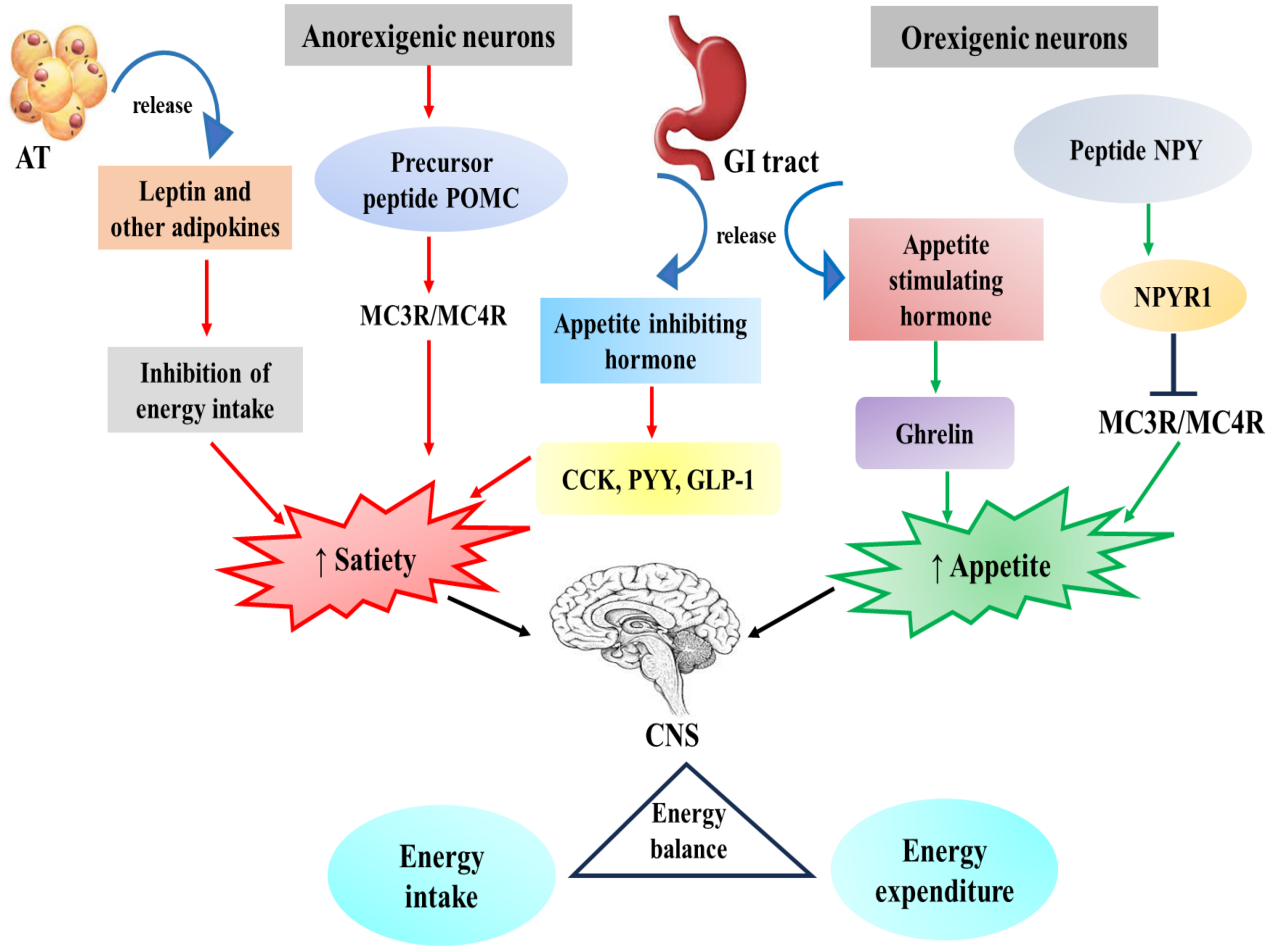
A summary of the ways that peripheral hormones regulate hunger and satiety in relation to orexigenic and anorexigenic stimuli. PYY, GLP‐1, and CCK are examples of endogenous gut hormones that the peripheral hormones use to control hunger and cause anorexia. In humans, ghrelin has an orexigenic action and increases appetite. Pointed arrows indicate activation; Blocked arrows indicate inhibition. AT, adipose tissue; CCK, cholecystokinin; GLP‐1, glucagon like peptide‐1; NPY, neuropeptide Y; POMC, propiomelanocortin; PYY, peptide YY; NPY, neuropeptide Y; CNS, central nervous system.

GLP‐1, also known as glucagon‐like peptide, is released by L‐cell enteroendocrine cells in the colon and small intestine in reaction to intraluminal nutrients, specifically fat and glucose. By promoting satiety and reducing calorie intake, GLP‐1 activates myenteric neurons and vagal afferent nerves, which lower the rate of gastric emptying (GE) and have anorexigenic effects. Similar effects are seen by other enteroendocrine‐produced peptides, such as cholecystokinin (CCK), which similarly slows GE, stimulates vagal afferents, and encourages meal termination (Makaronidis & Batterham, 2019). Insulin and amylin are released together by pancreatic beta cells. According to Li et al. (2015), it is additionally produced in the lateral hypothalamus, where it works in concert with leptin to decrease calorie intake. Aside from its effect on leptin activity, amylin also raises energy expenditure and affects hedonic elements of eating, which could result in choosing a particular meal type (Whiting et al., 2017) (Figure 3).

## 4 ROLE OF AT IN OBESITY

Adipocytes, preadipocytes, endothelial cells, and immune cells are among the various cell types found in AT, which is the primary and significant location for the storage of surplus energy in the form of triglycerides. It has been determined that there are three main varieties of AT: beige, brown adipose tissue (BAT), and WAT. WAT is known as the main energy reservoir of our body and responsible for secretes a huge number of hormones and cytokines that regulate metabolism and insulin resistance. Moreover, BAT, specialized in energy expenditure through no shivering thermogenesis via the mitochondrial uncoupling protein1 (UCP‐1) (Mathieu et al., 2010) (Table 1).

**TABLE 1:** Characteristics of white, brown, and beige adipocytes.

Adipocytes	Lipid droplets	Mitochondrial density	Deposition	UCP1 expression	Secreted factors	Function	Changes occur during obesity
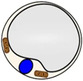 White adipose tissue	Large unilocular	Low	It is extensively spread throughout the body's viscera and subcutaneous tissue	Negative	Leptin, adiponectin, resistin	Lipogenesis, lipolysis, glucose uptake, adipokine secretion	Hyperplasia, hypertrophy, immune cell, infiltration, secretion of vasoconstrictors
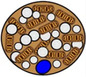 Brown adipose tissue	Small multilocular	High	It is mainly distributed in the interscapular region	Positive	FGF‐21, IL‐6	Thermogenesis, lipid clearance, glucose uptake, batokine secretion	Potentially resistant to obesity induced inflammation
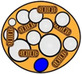 Beige	Small multilocular	High	In adults, it's beneath the skin close to the collarbone and spine	Positive	FGF‐21, IL‐6, SLIT‐2	Thermogenesis, catabolism	Loss of UCP1 expression

**FIGURE 4:**
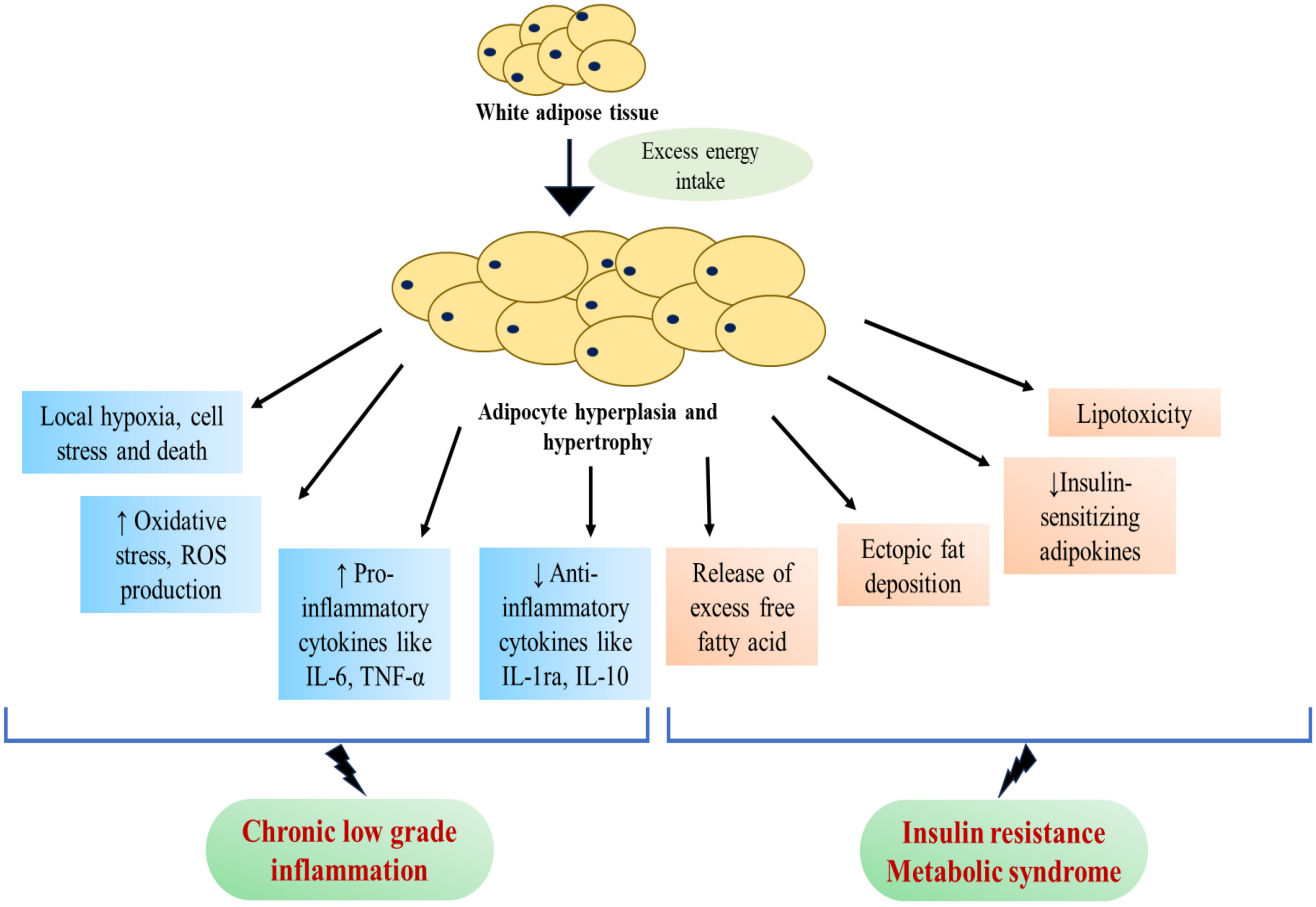
Adipocytokines and metabolic inflammation in adipose tissue. The interplay between immune cells and adipocytes that leads to AT malfunction produces adipocytokines, which are produced from AT. Obese patients frequently have changed levels and actions of adipocytokines, which can lead to diseases generated by obesity.

Basically, AT dysfunction is accumulation of ectopic fat including visceral obesity or central obesity (a surplus of fat accumulated in the abdomen, especially from excess visceral fat, is designated as central obesity), which is further characterized by changes in the cellular composition, increased lipid storage and impaired insulin sensitivity in adipocytes, and secretion of a proinflammatory, atherogenic, and diabetogenic adipokine pattern (Blüher et al., 2005). Obesity is the propagation form of WAT with the help of lipolysis process WAT increases release of FFAs that leads to raise of serum fatty acid levels. This over‐abundance of lipids has been considered a major reason for obesity‐associated insulin resistance and hepatosteatosis (Samuel et al., 2010) (Figure 4).

## 5 ROLE OF ADIPOCYTOKINES IN OBESITY AND OBESITY‐INDUCED METABOLIC DISORDERS

Leptin play important role to regulate hepatic lipogenesis by suppressing the expression of key enzymes in the fatty acid synthesis pathway (Cohen et al., 2002). AMP‐activated protein kinase (AMPK) is a crucial cellular energy sensor that is activated by leptin, which enhances muscle fatty acid oxidation (Minokoshi et al., 2002). In obesity, metabolic stress is diminished by protective role of adiponectin through its anti‐inflammatory effects. Tumor necrosis factor‐alpha (TNF‐α) production in obese mice is inhibits by adiponectin. Low levels of plasma adiponectin are associated with C‐reactive protein in humans (Ouchi et al., 2003). Adiponectin levels are negatively correlated with the adiposity and fasting glucose. The main role of TNF‐α is promotes insulin resistance of obesity and non‐insulin dependent diabetes mellitus (NIDDM). Specifically, in muscle and fat tissues insulin sensitivity improves through neutralization of TNF‐α by enhancing the activity of the insulin receptor tyrosine kinase (Heir & Stellwagen, 2020). Studies stated that increasing circulating level of resistin increases the risk of type 2 diabetes, inflammatory markers, atherosclerosis (Chen et al., 2009). IL‐6 is one of the major pro‐inflammatory cytokines as well as an anti‐inflammatory myokine, in obese patients IL‐6 serum level was found to be increased that resulting the development of chronic inflammatory conditions and serum lipid concentrations abnormalities (Galcheva et al., 2011). According to Blüher et al. (2005), IL‐10 is inversely linked with BMI and fasting blood sugar levels. Low levels of IL‐10 are associated with both the metabolic syndrome and T2D. Fatty acid synthase (FAS) is a central enzyme in lipogenesis which identified as a candidate gene for determining body fat. Sterol regulatory element binding protein‐1c (SREBP‐1c) is a member of a family of transcription factors that was initially engaged in the control of genes by the cellular availability of cholesterol (Sewter et al., 2002). Main function of lipoprotein lipase (LPL) is hydrolysis of triglyceride in chylomicron and VLDL and clearance of plasma triglyceride rich particles (Gonzales & Orlando, 2007) those are used for metabolic energy or re‐esterified into triglyceride which stored in AT. Moreover, peroxisome proliferator‐ activated receptors alpha (PPAR‐α) reduces endogenous fatty acid synthesis by blocking enzymes like acetyl‐CoA carboxylase and FASN. Ghrelin has various physiological functions, such as the stimulation of growth hormone release and of appetite, and fat accumulation. Ghrelin is the only peripheral hormone to transmit satiety signal (Sato et al., 2014).

## 6 BASIC COMPOSITION OF MARINE FO AND SYNTHESIS OF PRESENT ESSENTIAL FATTY ACIDS (N‐3 FATTY ACIDS AND N‐6 FATTY ACIDS)

**FIGURE 5:**
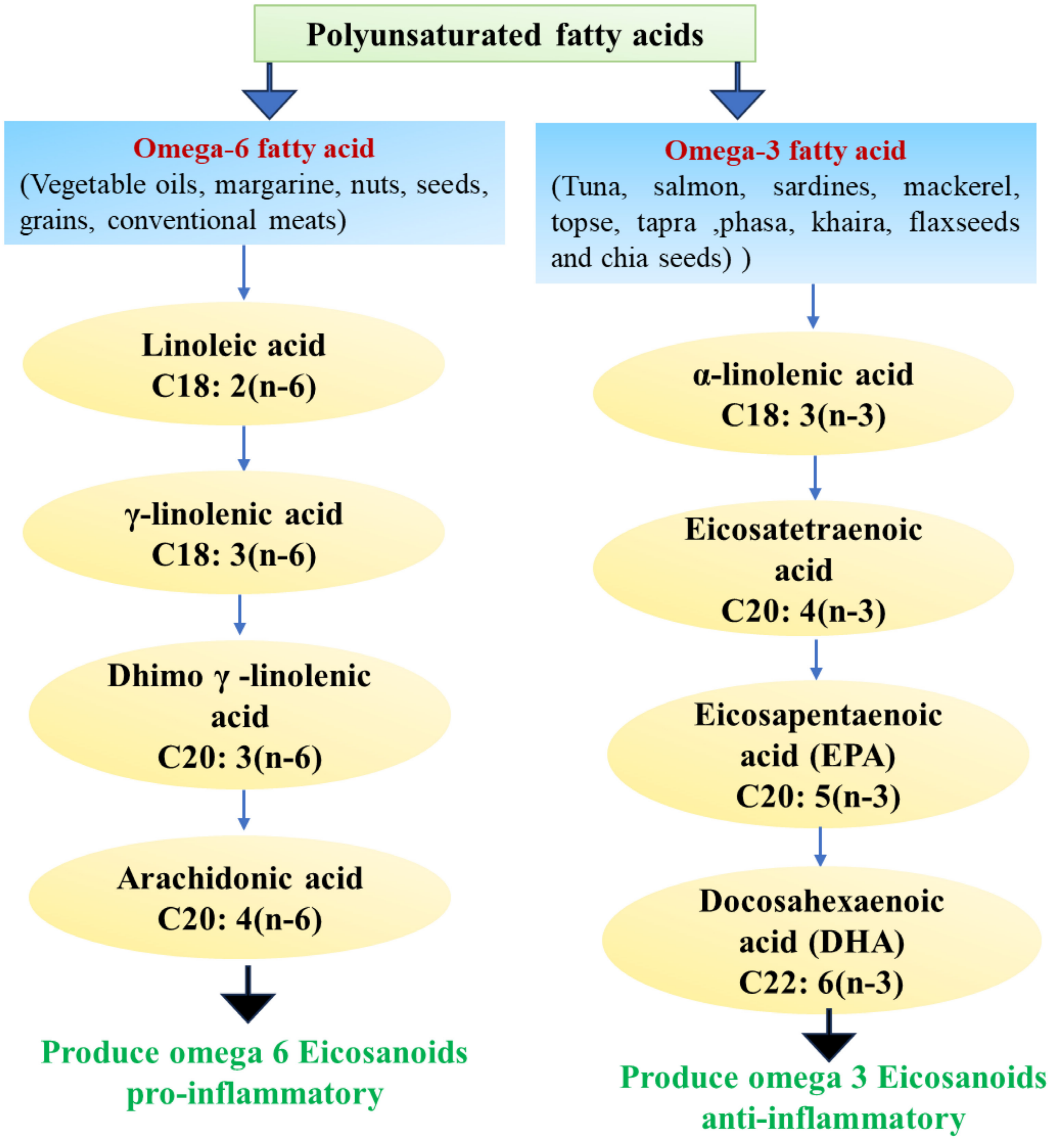
Classification of LC‐PUFAs. PUFAs are subdivided into two subclasses: n‐6 and n‐3 PUFAs. This flowchart represents general pathway of LC n‐3 and n‐6 fatty acids synthesis in human body. AA and proinflammatory eicosanoids are produced by the metabolism of LA, an important LC n‐6 PUFA. The necessary LC n‐3 PUFA ALA is broken down into EPA and DHA. Though they are thought to be more anti‐inflammatory, eicosanoids produced from the metabolism of EPA and DHA also help to regulate inflammation.

LC n‐6 PUFAs and LC n‐3 PUFAs are the two major PUFA families that are important to human health. Within most diets, the PUFAs with the largest concentrations are α‐linolenic acid (ALA, 18:3 n‐3) and linoleic acid (LA, 18:2 n‐6) (Djuricic & Calder, 2021). LA and ALA are essential fatty acids that cannot be synthesized by the human body and must be taken from outside (Al‐Khalaifah & Al‐Nasser, 2019). The LC n–3 PUFAs EPA, 20:5 n‐3, and DHA, 22:6 n‐3 can be synthesized very slowly from ALA in the human body with very little amounts (Kocatepe & Turan, 2018). For this reason, we need to consume foods rich in these oils in order to ensure that enough EPA and DHA are synthesized from our body. Additionally, vitamins A and D, which are essential for children's growth and development, are typically present in marine FO (Kaur et al., 2014). Marine FO provides micronutrients comprising selenium, iodine, potassium also. The European Food Safety Authority (EFSA) states that the ratio of LC n‐3 to LC n‐6 fatty acids ranges from 1.3 to 21.2. The balance of n‐6/n‐3 LC‐PUFAs is an important determinant in homeostasis maintenance, normal development, and mental health throughout the life cycle. Therefore, eating more fish may significantly improve the body's ability to absorb LC n‐3 fatty acids and maximize the use of EPA and DHA, which will benefit numerous physiological processes (Das et al., 2024). In order to keep the human body healthy, it is also crucial to take into account the ratio of dietary n‐6 to n‐3 fatty acids. However, the ratio of n‐6: n‐3 in the typical western diet is 10–20:1. A high n‐6/n‐3 ratio and excessive n‐6 PUFA consumption have been linked to an increased risk of several diseases, such as cancer, autoimmune diseases, obesity, inflammation, and cardiovascular disease (CVD). Conversely, a low n‐6/n‐3 ratio or an increase in high n‐3 PUFA intake have a suppressive impact. The ideal ratio may vary depending on the disease, but usually a 3:1–4:1 ratio is suggested to prevent many pathological diseases brought on by the modern western diet. For instance, a 4/1 ratio is linked to a 70% decrease in overall CVD mortality. The 2.5/1 ratio may inhibit rectal cell proliferation in patients with colorectal cancer. A lower n‐6/n‐3 ratio was linked to a lower risk in breast cancer‐affected women, and a 2–3/1 ratio was shown to be effective in reducing patients' obesity and its related inflammation (Kartikasari et al., 2024). Many studies have found low conversion rates of ALA to EPA and DPA, and little to no DHA synthesis; hence, any direct benefits of these very long‐chain fatty acids depend on dietary intake (Pawlosky et al., 2001). Both dietary intake and fatty acid desaturase activity determine plasma n‐3 PUFA levels (Vessby, 2003) (Figure 5).

## 7 ROLE OF MARINE FO ON OBESITY AND OBESITY‐ASSOCIATED METABOLIC DISORDERS

**FIGURE 6:**
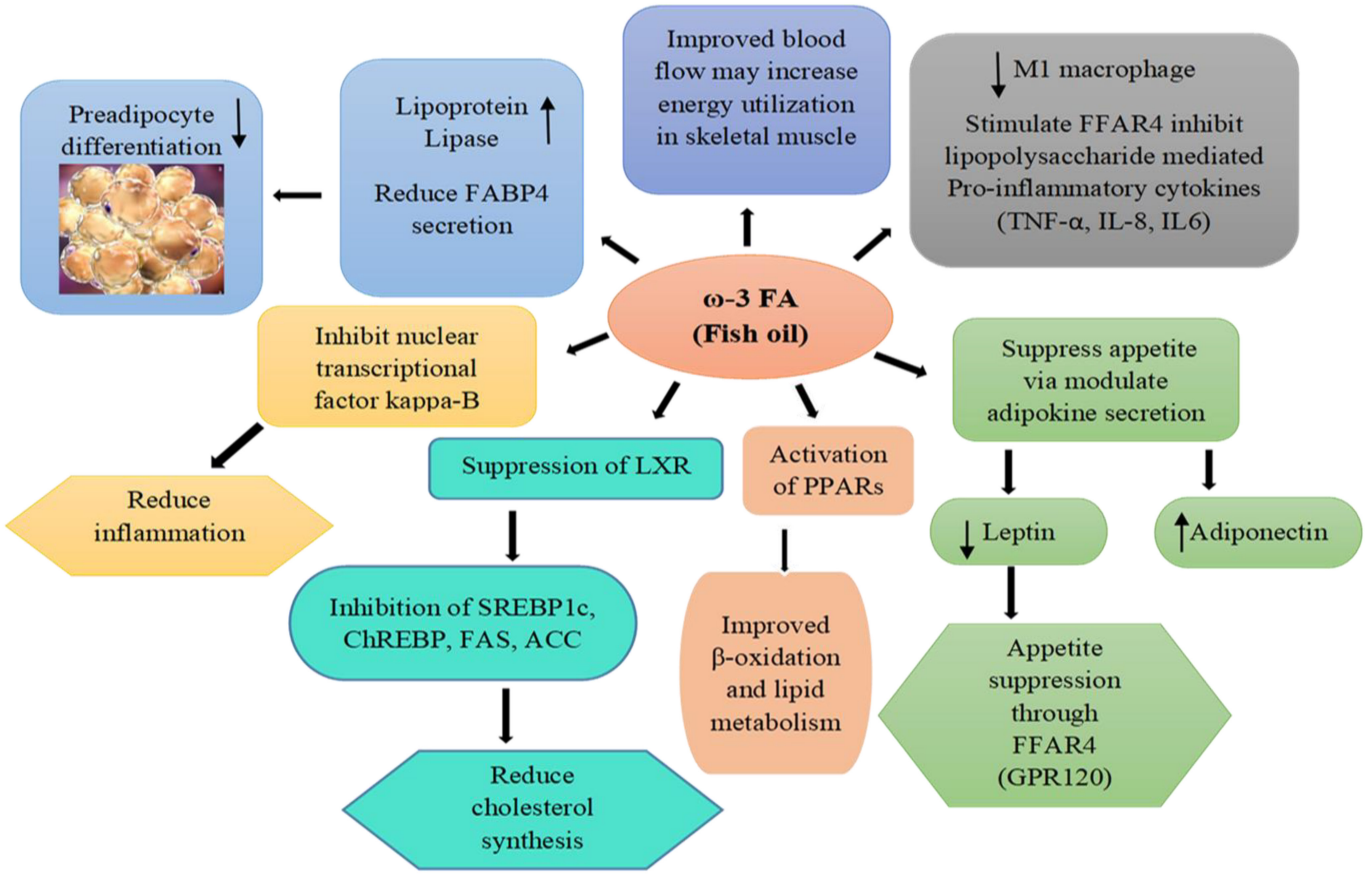
Beneficial effects of marine FO on obesity and obesity‐associated metabolic disorders. Marine FO containing n‐3 PUFAs can modulate adipokine secretion by suppressing the function of LXR, FABP4, SREBP‐1c, FAS, ChREBP. Eventually, inflammatory cytokines are expressed resulting in chronic inflammatory conditions in adipocytes of WAT. Dietary marine FO may have preventive effects on these events, leading to health benefits.

Recently marine FO has attracted special attention for its fruitful health beneficial effect. Marine FO exerts many defensive properties against several diseases such as obesity, cardiovascular diseases, rheumatoid arthritis, depression, cognitive decline, and neurological disorders, such as Alzheimer's, thought to have anti‐thrombotic and anti‐ inflammatory properties (Hostalek et al., 2015). Daily intake of fish through diet can diminish the risk of coronary heart disease for the presence of protective components such as EPA and DHA (Iso et al., 2006). LC n‐3 PUFAs are major ligands of PPARs. Their binding induced transcription of specific genes encoding inflammation in AT. Marine FO play important role to activated PPARα (Wang et al., 2021). β‐Oxidation is a catabolic process in myocytes that enhanced by EPA as well as it can responsible for ameliorate intramyocellular triacylglycerol lowering associated with compression of lipogenesis (Yu et al., 2011). DHA is incorporated from dietary marine FO in the hippocampus, where it controls the expression of genes implicated in the prevention of neurological diseases and can slow the age‐related cognitive decline (Denis et al., 2013). Consumption of dietary marine FO can play a significant impact in ghrelin modulation, which may affect feeding behavior and energy intake. Saidpour et al. (2012) found that a high‐fat meal combined with marine FO had the strongest stimulatory effect on fasting ghrelin expression and plasma level. However, Cao et al. (2022) investigated whether marine FO containing DHA could reduce diet‐induced insulin resistance in obese individuals, in part by regulating gut microbiome (e.g., lactobacillus) and promoting colonic PYY expression (via the mediation of short‐chain fatty acid receptor FFA4 in colon), both of which play important roles in linking diet, gut microbiome, and host metabolism. Research suggests that consuming natural fatty acids, such as marine FO (tuna, sardine, mackerel, and tapra), may help reduce obesity (Das et al., 2009). However, in order to support the current findings and explore the long‐term benefits of n‐3 fatty acid supplementation in managing overweight and obesity, a large‐scale, multi‐center, placebo‐controlled long‐term trial should be carefully planned. The researchers found that n‐3 PUFA supplementation could serve as an effective approach to lower triglyceride levels and waist circumference (Zhang et al., 2017). It has been suggested that tapra FO (a marine fish found in the Bay of Bengal) supplementation may reduce levels of body weight, BMI, serum total cholesterol, triglycerides, low‐density lipoprotein (LDL), and very‐low‐density lipoprotein (VLDL) in obese mice model where the mice was treated with 12.5 mg/kg body weight /day amount of tapra FO for 4 weeks of total experiment. However, marine FO contains various essential fatty acids which may increase lipid oxidation, decrease lipid synthesis, and inhibit lipogenesis (Pradhan et al., 2020). WAT produces leptin, which binds to brain receptors such as anandamide and neuropeptide Y (both of which are appetite promoters), reducing hunger and inducing satiety. In this way, it communicates with the brain to regulate food and energy consumption (Al‐Hussaniy et al., 2021). Panchali et al. (2024) explored that marine FO(treated with Phasa FO, 12.5 mg/kg body weight/day) containing conjugated fatty acids (specially LC n‐3 PUFAs) can inhibit the protein and mRNA expression of leptin by correlated with the inhibition of fat accumulation and adipocyte proliferation due to disruption of leptin signaling (Figure 6). According to US Food and Drug Administration guidelines, including up to 3 gm of marine FO/day in the diet is generally considered safe. Increased bleeding time and drug interactions are two possible side effects that may be concerning at larger doses, but these symptoms are usually not regarded as clinically significant (Fetterman Jr & Zdanowicz, 2009).

## 8 MECHANISM OF N‐3 PUFAS IMPROVE ADIPOSITY AND OBESITY THROUGH LIPID METABOLISM VIA THE PPAR‐ γ AND AMPK PATHWAYS

**FIGURE 7:**
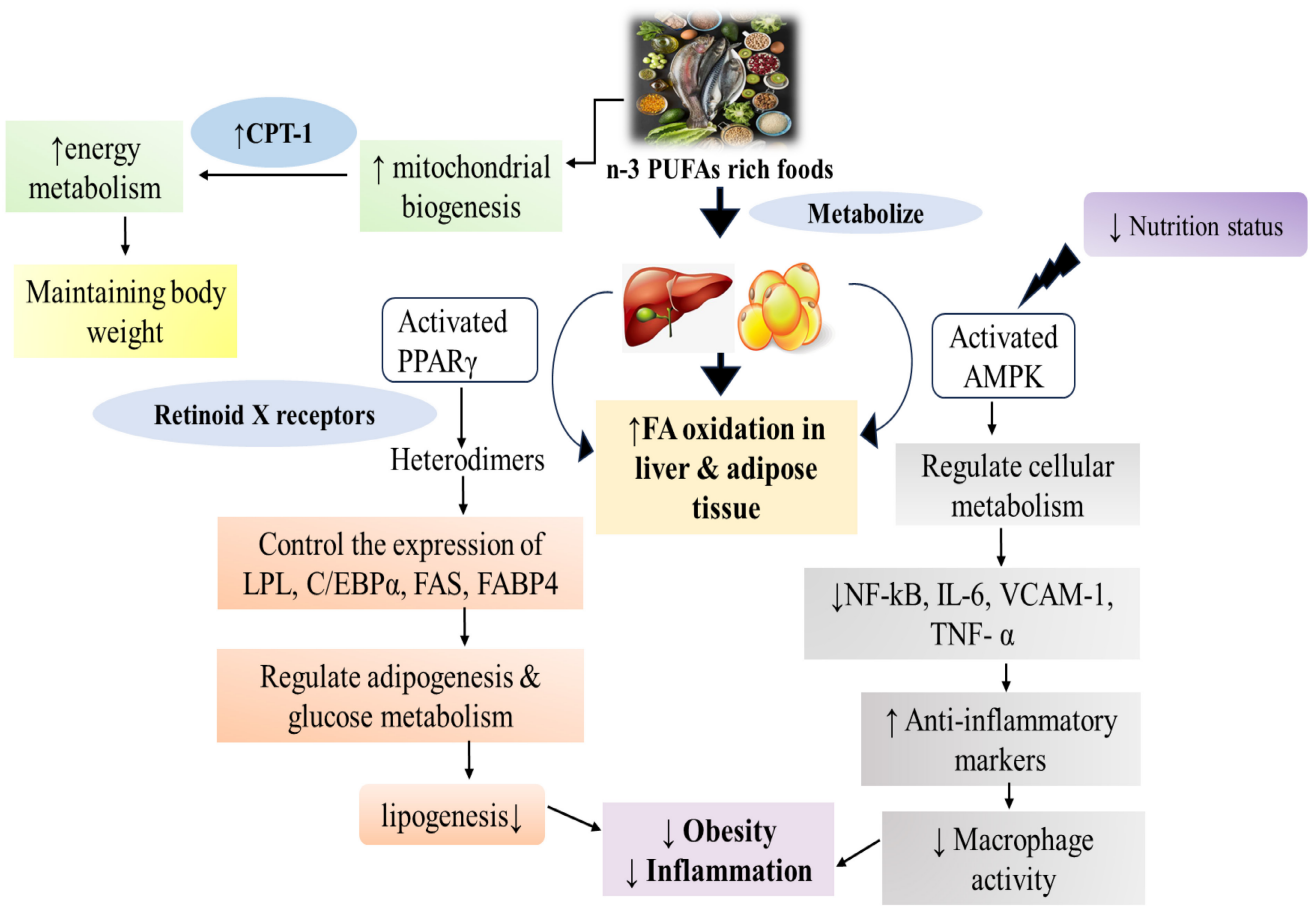
Mechanisms mediating effects of n‐3 PUFA on liver and AT metabolism. LC n‐3 PUFA increase fatty acid oxidation in the liver and adipose tissue, thus limiting fat storage in these tissues. LC n‐3 PUFA also decrease the production and release of pro‐inflammatory adipokines by two major pathways PPAR‐ γ and AMPK pathways. All mechanisms depicted here contribute to an improved metabolic profile.

There are several proposed mechanisms by which n‐3 PUFA could work in reducing body weight and improving the metabolic profile. AT expansion in obesity occurs via adipocyte hypertrophy (enlargement of adipocytes) and hyperplasia (increase in adipocyte number due to adipogenesis). LC n‐3 PUFAs alter expression and nuclear localization of both the transcription factor SREBP‐1 and the ChREBP, which control several lipogeneic genes, including those regulating cholesterol and fatty acid synthesis. Furthermore, n‐3 PUFAs suppress hepatic lipogenesis by reducing both messenger RNA (mRNA) and active protein expression of SREBP‐1c, which results in reduced expression of many genes involved in lipogenesis, including FAS and acetyl‐coA carboxylase. PPARs are transcription factors that form heterodimers with retinoid X receptors in the promoter regions of several genes involved in lipid and glucose metabolism. For example, LC n‐3 PUFA activation of PPARα decreases lipogenesis by suppressing FAS activity (Kaur et al., 2011). It has been suggested that PPARγ plays a significant role in the ability of n‐3 PUFA, specifically DHA, to stimulate M2 macrophage polarization and thereby reduce inflammation. LC n‐3 PUFAs have been shown to increase mitochondrial biogenesis and fatty acid oxidation in the liver, AT and small intestine of rodents, possibly through PPARα and COX3 induction (Hensler et al., 2011). PUFA‐controlled genes involved in lipid oxidation and thermogenesis include mitochondrial HMG‐CoA synthase, peroxisomal acyl‐CoA oxidase, hepatic CPT‐1, FABP and fatty acid transporter proteins. Activation of PPARα can also increase fatty acid oxidation. Increases in fatty acid oxidation by n‐3 PUFA may also be mediated by AMPK, a known regulator of cellular energy metabolism. AMPK upregulation by n‐3 PUFA has been proved in both AT and cultured adipocytes. As a central energy sensor, the AMPK has now been explored to play a paramount role in fat synthesis and catabolism, especially in regulating the energy expenditure of brown/beige adipose tissue and the browning of WAT (Kasbi Chadli et al., 2012) (Figure 7).

## 9 FUTURE TRENDS

According to the present assessment, marine FO is an useful productive field of research with bright future prospects. Thus, by using FO as the only source of dietary oil and varying the amount or type of oil in the diet, marine fish can be readily farmed to yield fillets with levels of oil, 22:6n‐3 and 20:5n‐3 as required to suit consumer demand and human nutritional and health requirements. Modem fish farming, and especially marine fish farming, has much to offer, whether nutritionally, economically, or ethically, for optimizing the exploitation of limited global marine resources of FO. On the basis of current situation, it has been predicted that during the decade to come, marine FO might be revolutionize for the management for multiple illnesses.

## 10 CONCLUSION

Marine FO has received a lot of attention in medical science over the last few decades, and some experts recommend it because of its functional properties. Marine FO is a growing source of LC n‐3 fatty acids, such as DHA and EPA, which can mitigate the negative effects of long‐term illnesses. This review work was done to look after how marine FO fight against obesity and obesity‐associated inflammation via several mechanism of LC n‐3 fatty acids. We may draw the conclusion that, in order to improve society, a greater portion of the populace needs to be made aware of the quantity, significance, and health impact of marine FO.

## AUTHOR CONTRIBUTIONS

**Riya Kar:** Writing—review, editing & preparing figures and table, Writing—original draft, visualization, resources, project administration, investigation, formal analysis. **Titli Panchali:** Writing—review & editing, formal analysis. **Pipika Das:** Writing—original draft, investigation, formal analysis. **Ananya Dutta** and **Manisha Phoujdar:** Performed formal analysis. **Shrabani Pradhan:** Conceptualized the study, supervised the review, editing and approved the final manuscript.

## FUNDING INFORMATION

This work was supported by own fundings, Biodiversity and Environmental Studies Research Center, Midnapore City College, Bhadutala, Paschim Medinipur, 721129, West Bengal, India.

## CONFLICT OF INTEREST STATEMENT

The authors declare that they have no conflicts of interest.

## ETHICS STATEMENT

This work does not involve trials on any human or animals.

## Data Availability

The data that support the findings of the study are available from the corresponding author upon request.
